# Social support and subjective assessment of psychophysical condition, health, and satisfaction with quality of life among women after pregnancy loss

**DOI:** 10.1186/s12884-021-04093-w

**Published:** 2021-11-05

**Authors:** Grażyna Iwanowicz-Palus, Mariola Mróz, Agnieszka Bień, Krzysztof Jurek

**Affiliations:** 1 Chair and Department of Development in Midwifery, Faculty of Health Sciences, Medical University of Lublin, 4-6 Staszica St., 20-081 Lublin, Poland; 2 Obstetrics and Gynecology Department and Clinic, Cardinal S. Wyszyński Regional Specialist Hospital in Lublin, Lublin, Poland; 3 Institute of Sociological Sciences, Faculty of Social Sciences, The John Paul II Catholic University of Lublin, Lublin, Poland

**Keywords:** Social support, Miscarriage, Ectopic pregnancy, Pregnancy loss, Psychophysical condition, Quality of life

## Abstract

**Background:**

The task of modern medicine is not just to heal, but also to improve the patient’s well-being and achieve non-medical goals in the therapy process that enable effective physical, mental and social functioning of the patient. Social support in difficult situations mobilizes an individual’s strength and resources to cope with problems. Research on social support and women’s condition after pregnancy loss reflects a holistic approach to the patient and is important from the perspective of increasing the level of hospital care.

**Objective:**

The aim of our study was to assess the impact of social support on the psychophysical condition, health, and satisfaction with quality of life among women after miscarriage and ectopic pregnancy.

**Methods:**

The cross-sectional study was carried out in a group of 500 patients after miscarriage and 110 with ectopic pregnancy, hospitalized in hospitals in Lublin (Poland). The study was conducted with the use of a diagnostic survey, comprising the Berlin Social Support Scales (BSSS) and an original survey questionnaire (psychophysical condition, satisfaction with health and quality of life on a scale of 1–4, sources of support on a scale of 1–10, with 1 being the poorest rating).

**Results:**

Respondents after miscarriage and those after ectopic pregnancy assigned the highest scores to the degree of perceived available instrumental support (respectively, miscarriage: M = 3.79, EP: M = 3.77). Women after pregnancy loss assigned the highest score to the support obtained from their partner (respectively, miscarriage: M = 9.26, EP: M = 9.23). Social support was significantly correlated with the condition of patients hospitalized as a result of pregnancy loss (*p* < 0.05). The assessment of psychophysical condition, health, and QoL of the respondents is determined by their education, financial standing, and obstetric history (*p* < 0.05).

**Conclusions:**

Women hospitalized due to miscarriage and ectopic pregnancy assigned high scores to the level of perceived available instrumental, emotional, and actually received social support. There is a positive relationship between social support and subjective opinion about psychophysical condition, health and satisfaction with quality of life among women after pregnancy loss. The assessment is determined by sociodemographic factors and the respondents’ obstetric history.

**Supplementary Information:**

The online version contains supplementary material available at 10.1186/s12884-021-04093-w.

## Background

Social support in difficult situations is understood as a special method and type of assistance, which mobilizes an individual’s strength and resources to cope with problems. It helps minimize stress related to a health situation, has a beneficial influence on various aspects of psychophysical well-being, and is associated with the overall assessment of quality of life (QoL) [1, 2].

The notion of support, understood in the broad sense, focuses on issues of social integration as well as sources and systems of support characterized by the existence of bonds, a sense of belonging, and relationships between people, affecting the individual and allowing them to feel that they are surrounded by people on whom they can rely [3, 4].

The research on social support in medical sciences reflects a holistic approach to the patient, a significant aspect of how patients and their families function. It is also important for improving the level of care provided by hospital staff [1, 2, 5].

Pregnancy loss is regarded as a critical event, as it is usually traumatic in nature. The consequence of this painful experience is mourning, which is a multidimensional process with mental, somatic, behavioral, and social aspects. A person in mourning requires comprehensive assistance, and social support plays an important role in moving through its successive stages [6, 7].

Improper behavior of the environment, inadequate support, or a complete lack thereof means that women cannot afford to fully process their negative emotions, which may lead to mental disorders (depression, anxiety, post-traumatic stress disorder – PTSD) and loss of confidence in medical personnel [8–10].

Unfortunately, in many countries, the main focus of clinical practice is the patient’s clinical condition and treatment, while the extrasomatic consequences are of secondary importance. It is also unfortunate that training for medical personnel in the field of pregnancy loss management and providing appropriate support is often absent. Scientific research shows that providing support in a professional manner, adapted to the expectations and needs of the patient, has an impact on their satisfaction with the care received. The actions of hospital staff also have very significant implications for the women’s immediate and long-term well-being. Therefore, the importance of non-medical goals in the therapy process is emphasized, including improvements in the patient’s well-being to enable effective functioning, not only physical but also mental and social [1, 11–13].

The main purpose of our study was to evaluate the social support received by women after pregnancy loss, and its impact on their subjectively reported overall psychophysical condition, health, and quality of life.

Specific objectives included answering the following research questions:

- Does subjectively reported psychophysical condition and support received differ between patients after pregnancy loss due to miscarriage and ectopic pregnancy?
- Are selected dimensions and sources of support correlated with subjectively reported psychological condition, health, and quality of life among women after pregnancy loss due to miscarriage and ectopic pregnancy?

Because of the significance and complexity of the issue of pregnancy loss, it is important to assess social support and its impact on the subjective assessment of the psychophysical condition and health of women affected by this problem as well as to conduct a comparative analysis between the different types of loss among women who have experienced a miscarriage or ectopic pregnancy, which is less frequently considered in non-clinical research. The research conducted will help determine the effectiveness of the support provided, facilitate the planning and implementation of care adjusted to the needs of this group of patients, and exert an influence on improving the quality of care for hospitalized women.

## Methods

### Data collection

The study was carried out from August 2016 to February 2019 among patients hospitalized in Lublin in medical units with the highest referral level of perinatal care. The study included 645 participants, with 610 correctly completed questionnaires being qualified for further statistical analysis (including 500 by women after miscarriage and 110 from respondents with ectopic pregnancy). The effectiveness index of this data was 94.57%.

Timing of the study — the survey questionnaire was given to each patient on the last day of her hospitalization, having ascertained that her treatment had been completed. This way, the patient could refer to all social support received throughout her hospitalization. Each patient received instructions on how to complete the questionnaire correctly, and completed the questionnaire herself. The inclusion criteria for the study were: consent to participate in the diagnostic survey, age over 18, diagnosis of a single pregnancy loss as a result of spontaneous abortion up to the 22nd week of the pregnancy or a diagnosis of ectopic pregnancy, normal clinical condition, no psychophysical disorders. The study excluded patients in a poor mental state or undergoing psychological therapy or psychiatric treatment. To avoid interference/confounders/situations that could affect the reliability of the study (e.g. administering the questionnaire to a patient under the influence of sedatives or anesthetics after a procedure etc.), information about each patients’ stage of treatment, time of hospitalization, psychological and physical condition was obtained from medical personnel before contacting the patient directly.

The independent variable in the study was social support, and dependent variables included psychophysical condition, health, and quality of life. Covariates included age, residence, relationship status, education, professional activity, self-reported financial standing.

The study was conducted with the use of a diagnostic survey. The research tool applied was a questionnaire consisting of two parts:

- ■ Berlin Social Support Scales (BSSS) R. Schwarzer, U. Schulz – a set of tools measuring specific dimensions of social support: perceived available, actually provided and received support, need for support, support seeking, and protective buffering. Respondents rate their agreement with the statements included in the questionnaire on a scale of 1–4, with 1 denoting strong disagreement and 4 strong agreement. The higher the score, the higher the intensity of social support. The scales are intended for use in the general population, and have been adapted into different language versions. The Cronbach’s α coefficient for this tool is 0.80 [14, 15].
- ■ Original survey questionnaire – this takes into account the characteristics of the women surveyed (age, residence, relationship status, education, professional activity, self-reported financial standing) and questions concerning the subject of the study (psychophysical condition, health, and support from each source). The respondents scored the level of support received from specific sources (obstetrician-gynecologist, midwife, psychologist, husband/partner, family, friends, clergy, patients in a similar situation staying at the hospital at the same time), marking their answers on a 10-point scale, where 1 meant that the support was completely insufficient and 10 completely sufficient. The respondents made a subjective assessment of their mental and physical condition, satisfaction with health and quality of life on a scale of 1–4, where 1 denoted “poor:, and 4 “very good”, (Supplementary files 1 and 2 ). The Cronbach’s α coefficient for this tool is 0.90.

### Statistical analysis

The collected research material was analyzed statistically using the IBM SPSS Statistics (version 21) statistical package. Quantitative variables were described by means of mean (M), median (Me), standard deviation (SD), as well as minimum (Min) and maximum (Max) values. For qualitative variables, the number (n) and percentage were provided for each category. The Mann-Whitney U test (Z) was used to compare two independent groups. The Chi-Square test of independence (*χ*^*2*^*) and the Spearman’s Rank Correlation Coefficient (rho) were used to determine the relationship between variables measured on a qualitative scale.* Multiple regression analysis was used to determine the statistically significant associations between mental condition, physical condition, health satisfaction, quality of life and independent variables (four models). For the purpose of this analysis, dummy coding was used for variables such as relationship status, education, having children, and history of pregnancy loss. Linearity assumptions and homogeneity of variances were checked with scatter plots, and there was no heteroscedasticity/no clear pattern on the plots. Skewness ranged between ±1. Multicollinearity was checked, and the minimum and maximum variable inflation factors (VIF) reported were 1.021 and 1.332, respectively, which indicates that there was no multicollinearity threat. The overall F-test and the adjusted R-squared were considered. Un-standardized Beta (B) and standardized Beta (β) coefficients were computed to assess the level of association and statistical significance in multiple regression analysis. The variables with a *P*-value < 0.05 were declared significantly associated with mental condition, physical condition, health satisfaction or quality of life.

### Ethical approval

The study was approved by the Bioethics Committee at the Medical University of Lublin (*KE-0254/221/2016*) and the executive boards of the medical units and the heads of the hospital departments where the research was conducted. The respondents were informed that their participation in the study was voluntary and anonymous, and that the results were used only for scientific purposes. Written consent to participate in the study was obtained from the patients after discussing its course and purpose.

## Results

Table 1 presents the characteristics of the women participating in the diagnostic survey. The study included 610 patients hospitalized as a result of pregnancy loss; 500 (82.0%) with miscarriage and 110 (18.0%) with ectopic pregnancy (EP). The group of respondents after miscarriage consisted predominantly of women aged 31–35 (32.4%), living in voivodship capital cities (50.2%), married (78.8%), with higher education (61.0%), performing intellectual work (48.6%), and considering their socio-economic standing to be good (61.2%). Furthermore, most of the women after miscarriage had planned their pregnancy (71.8%), had had children previously (59.4%), and had miscarried for the first time (59.4%). On the other hand, the majority of the respondents diagnosed with ectopic pregnancy were women between 26 and 30 years of age (35.5%), living in the countryside (40.9%), married (88.2%), with higher education (61.8%), performing intellectual work (49.1%), and considering their socio-economic standing to be good (59.1%). Furthermore, most of the women had planned their pregnancy (66.4%), had had children previously (57.3%), and had pregnancy loss for the first time (58.2%), (Table 1).

**Table 1:** Participants’ characteristics

Participants’ Characteristics		Miscarriage		EP	
		n	*%*	n	%
Age	< 25 y/o	65	13.0	12	10.9
	26–30 y/o	160	32.0	39	35.5
	31–35 y/o	162	32.4	33	30.0
	> 35 y/o	113	22.6	26	23.6
Residence	Urban – province capital	251	50.2	40	36.4
	Urban – other	91	18.2	25	22.7
	Rural	158	31.6	45	40.9
Relationship status	Married	394	78.8	97	88.2
	Single	106	21.2	13	11.8
Education	Other than college/university	195	39.0	42	38.2
	College/university	305	61.0	68	61.8
Professional activity	Professionally inactive	117	23.4	18	16.4
	White-collar work	243	48.6	54	49.1
	Blue-collar work	140	28.0	38	34.5
Self-reported financial standing	Very good	80	16.0	13	11.8
	Good	306	61.2	65	59.1
	Moderate	112	22.8	32	29.1
Having children	Yes	297	59.4	63	57.3
	No	203	40.6	47	42.7
Planned pregnancy	Yes	359	71,8	73	66.4
	No	141	28.2	37	33.6
History of pregnancy loss	Yes	203	40.6	46	41.8
	No	297	59.4	64	58.2

*EP* ectopic pregnancy

Both the respondents after miscarriage and ectopic pregnancy assigned high scores to the degree of perceived available instrumental support (respectively, miscarriage: M = 3.79, EP: M = 3.77), emotional support (M = 3.68, M = 3.65), and actually received support (M = 3.61, M = 3.57). Their need for support and support-seeking were less intense. There were no statistically significant differences between the groups studied in their assessment of social support (*p* > 0.05).

Women after miscarriage and women after ectopic pregnancy assigned the highest score to the support obtained from their partner (respectively, miscarriage: M = 9.26, EP: M = 9.23), family (M = 9.09, M = 9.18), midwife (M = 8.70, M = 8.58), and friends (M = 8.49, M = 8.61), and then from the obstetrician-gynecologist (M = 8.03, M = 7.74). The clergy (M = 5.57, M = 5.92) and psychologists (M = 3.97, M = 3.33) constituted a smaller source of support for the hospitalized patients. There was no correlation between the groups (*p* > 0.05) in their assessment of support obtained from specific sources.

Women after miscarriage and women after ectopic pregnancy assigned the highest scores to their overall quality of life (respectively, miscarriage: M = 2.93, EP: M = 2.81), while the lowest scores was assigned to satisfaction with their overall health (M = 2.62, M = 2.39) and mental condition (M = 2.68, M = 2.63). Data analysis showed that women after miscarriage statistically significantly more often (*p* < 0.001) expressed satisfaction with their overall health (M = 2.62) when compared to women with ectopic pregnancy (M = 2.39), (Table 2).

**Table 2:** Assessment of the dimensions and sources of social support and the subjective assessment of psychophysical condition, health, and quality of life among women after miscarriage and ectopic pregnancy

	Variables		M	SD	Me	Min	Max	S**tatistics**
**Social support (BSSS)**^**A**^	Perceived available emotional support	Miscarriage	3.68	0.45	3.75	1.00	4.00	Z = −0.705 *p* = 0.481
		EP	3.65	0.43	3.75	2.50	4.00	
	Perceived available instrumental support	Miscarriage	3.79	0.43	4.00	1.00	4.00	Z = −0.218 *p* = 0.827
		EP	3.77	0.43	4.00	2.00	4.00	
	Need for support	Miscarriage	3.17	0.56	3.25	1.00	4.00	Z = −0.490 *p* = 0.624
		EP	3.14	0.60	3.25	1.50	4.00	
	Support seeking	Miscarriage	3.10	0.66	3.20	1.00	4.00	Z = −0.311 *p* = 0.756
		EP	3.07	0.69	3.00	1.00	4.00	
	Actually received support	Miscarriage	3.61	0.40	3.80	1.00	4.00	Z = −1.634 *p* = 0.102
		EP	3.57	0.39	3.73	1.40	4.00	
	Protective buffering	Miscarriage	1.87	0.68	1.83	1.00	4.00	Z = −1.668 *p* = 0.095
		EP	1.98	0.64	2.00	1.00	4.00	
**Sources of support**^**B**^	Gynecologist	Miscarriage	8.03	2.43	9.00	1.00	10.00	Z = −1.660 *p* = 0.097
		EP	7.74	2.41	8.00	1.00	10.00	
	Midwife	Miscarriage	8.70	1.93	10.00	1.00	10.00	Z = −0.786 *p* = 0.432
		EP	8.58	2.05	9.00	1.00	10.00	
	Psychologist	Miscarriage	3.97	3.73	1.00	1.00	10.00	Z = −1.257 *p* = 0.209
		EP	3.33	3.47	1.00	1.00	10.00	
	Partner	Miscarriage	9.26	2.03	10.00	1.00	10.00	Z = −1.930 *p* = 0.054
		EP	9.23	1.87	10.00	1.00	10.00	
	Family	Miscarriage	9.09	2.07	10.00	1.00	10.00	Z = −0.861 *p* = 0.389
		EP	9.18	1.71	10.00	1.00	10.00	
	Friends	Miscarriage	8.49	2.66	10.00	1.00	10.00	Z = −0.423 *p* = 0.672
		EP	8.61	2.33	10.00	1.00	10.00	
	Member of the clergy	Miscarriage	5.57	3.84	5.00	1.00	10.00	Z = −0.729 *p* = 0.466
		EP	5.92	3.49	5.00	1.00	10.00	
	Patients at hospital after pregnancy loss	Miscarriage	7.80	3.03	9.00	1.00	10.00	Z = −0.635 *p* = 0.525
		EP	7.76	2.80	9.00	1.00	10.00	
**Psychophysical condition**^**C**^	Mental condition	Miscarriage	2.68	0.83	3.00	1.00	4.00	Z = −0.874 *p* = 0.382
		EP	2.63	0.75	3.00	1.00	4.00	
	Physical condition	Miscarriage	2.85	0.65	3.00	1.00	4.00	Z = −0.803 *p* = 0.422
		EP	2.78	0.72	3.00	1.00	4.00	
	Health satisfaction	Miscarriage	2.62	0.71	3.00	1.00	4.00	Z = −3.268 *p* = 0.001
		EP	2.39	0.72	2.00	1.00	4.00	
	Quality of life	Miscarriage	2.93	0.72	3.00	1.00	4.00	Z = −1.915 *p* = 0.055
		EP	2.81	0.70	3.00	1.00	4.00	

*EP* ectopic pregnancy, *M* mean, *SD* standard deviation, *Me* median, *Min* minimum*, Max* maximum

^A^Assessment of social support by BSSS

^B^ Level of social support received from specific sources of support during hospitalization

^C^ Subjective assessment of the psychophysical condition, health, and quality of life of women

Data analysis showed statistically significant (*p* < 0.05) positive correlations between mental condition and support provided to patients with ectopic pregnancy by the gynecologist-obstetrician (rho = 0.346), psychologist (rho = 0.327), and partner (rho = 0.231). On the other hand, in the case of women after miscarriage, mental condition significantly (*p* < 0.05) positively correlated with support from the family (rho = 0.104). Among women with EP, a statistically significant positive (*p* < 0.05) relationship was reported between satisfaction with health and support from the midwife (rho = 0.229), doctor (rho = 0.226), and friends (rho = 0.232), while among respondents who had miscarried, satisfaction with health positively correlated with support from the clergy (rho = 0.128). Quality of life in both groups of respondents showed a statistically significant (*p* < 0.05) positive correlation with support received from their relatives (rho range = 0.130–0.328), gynecologist-obstetrician (respectively: miscarriage: rho = 0.132, EP: rho = 0.195), midwife (rho = 0.137, rho = 0.198), and the clergy (rho = 0.180, rho = 0.264). Support from other patients in a similar situation significantly (*p* = 0.001) positively correlated in the group of patients after miscarriage (rho = 0.182), (Table 3).

**Table 3:** Analysis of correlations between the support obtained from specific sources and the subjective assessment of psychophysical condition, health, and quality of life among women after miscarriage and ectopic pregnancy

Sources of support		**correlations**							
		Mental condition		Physical condition		Health satisfaction	Quality of life		
		rho	*p*	rho	*p*	rho	*p*	rho	*p*
Gynecologist	Miscarriage	0.023	0.613	0.013	0.779	0.003	0.947	0.132	0.003
	EP	0.346	< 0.001	0.133	0.166	0.226	0.017	0.195	0.041
Midwife	Miscarriage	0.059	0.200	0.059	0.194	0.089	0.052	0.137	0.003
	EP	0.178	0.063	0.098	0.310	0.229	0.016	0.198	0.039
Psychologist	Miscarriage	0.003	0.966	0.107	0.097	0.089	0.168	0.002	0.974
	EP	0.327	0.026	0.019	0.901	−0.022	0.884	−0.033	0.827
Partner	Miscarriage	0.037	0.416	−0.003	0.943	− 0.019	0.681	0.130	0.004
	EP	0.231	0.016	0.102	0.291	0.180	0.061	0.328	< 0.001
Family	Miscarriage	0.104	0.024	0.051	0.270	0.018	0.701	0.175	< 0.001
	EP	0.121	0.211	0.095	0.327	0.088	0.362	0.201	0.036
Friends	Miscarriage	0.087	0.068	0.013	0.783	0.007	0.885	0.151	0.001
	EP	0.119	0.226	0.129	0.189	0.232	0.017	0.261	0.007
Member of the clergy	Miscarriage	0.094	0.115	0.065	0.273	0.128	0.031	0.180	0.002
	EP	0.029	0.821	−0.083	0.512	0.052	0.680	0.264	0.033
Patients at hospital after pregnancy loss	Miscarriage	0.088	0.077	0.021	0.680	0.064	0.197	0.182	< 0.001
	EP	0.071	0.507	0.183	0.084	−0.049	0.644	0.168	0.112

*EP* ectopic pregnancy

The regression analysis performed demonstrated that higher scores assigned to mental condition were associated with unplanned pregnancy that ended in pregnancy loss (ß = − 0.147, *p* < 0.001), better financial standing (ß = − 0.245, *p* < 0.001) and higher level of received social support (ß = 0.145, *p* < 0.001).

Physical condition was positively associated with financial standing (ß = − 0.177, *p* < 0.001) and received social support (ß = 0.108, *p* = 0.009).

Satisfaction with health was associated with having children (ß = 0.185, *p* < 0.001), pregnancy loss due to miscarriage (ß = 0.177, *p* < 0.001), better financial standing (ß = − 0.139, *p* < 0.001) and received social support (ß = 0.124, *p* < 0.002).

Higher QoL among the women studied was associated with having children (ß = 0.106, *p* = 0.015), no history of pregnancy loss (ß = 0.135, *p* = 0.001), better financial standing (ß = − 0.240, *p* < 0.001), higher education (ß = 0.081, *p* = 0.046) and received social support (ß = 0.222, *p* < 0.001) (Table 4).

**Table 4:** Multiple regression analysis models showing factors independently associated with mental condition, physical condition, health satisfaction or quality of life among women after miscarriage and ectopic pregnancy

Predictor	Mental condition			Physical condition			Health satisfaction			Quality of life		
	B	Beta	*p*	B	Beta	*p*	B	Beta	*p*	B	Beta	*p*
Constant	2.279		< 0.001	1.702		< 0.001	1.977		< 0.001	1.782		< 0.001
Having children^A^	0.104	0.063	0.159	−0.038	−0.028	0.542	0.285	0.185	< 0.001	0.154	0.106	0.015
History of pregnancy loss	0.007	0.004	0.915	0.048	0.035	0.415	0.118	0.076	0.070	0.198	0.135	0.001
Planned pregnancy^B^	−0.264	−0.147	< 0.001	− 0.002	− 0.002	0.968	− 0.087	− 0.053	0.199	0.007	0.005	0.908
Cause of pregnancy loss^C^	0.043	0.020	0.602	0.043	0.025	0.540	0.349	0.177	< 0.001	0.093	0.050	0.191
Age	−0.004	−0.005	0.898	−0.004	− 0.007	0.873	− 0.013	−0.021	0.625	0.002	0.004	0.930
Self-reported financial standing	−0.312	−0.245	< 0.001	− 0.184	− 0.177	< 0.001	− 0.164	−0.139	< 0.001	− 0.269	− 0.240	< 0.001
Relationship status^D^	0.107	0.052	0.221	−0.026	−0.016	0.721	−0.070	− 0.036	0.392	0.091	0.050	0.224
Education^E^	0.031	0.019	0.650	−0.055	− 0.040	0.349	0.032	0.020	0.622	0.119	0.081	0.046
Received support (BSSS)	0.275	0.145	< 0.001	0.166	0.108	0.009	0.217	0.124	0.002	0.369	0.222	< 0.001
	*F* = 8.587, *p* < 0.001 *R*^*2*^ = 0.12			*F* = 3.504, *p* < 0.001 *R*^*2*^ = 0.05			*F* = 7.882, *p* < 0.001 *R*^*2*^ = 0.11			*F* = 11.941, *p* < 0.001 *R*^*2*^ = 0.14		

B - Un-standardized Beta coefficient; β = standardized Beta coefficient; *p* – *p*-value; F –F-test for linear regression; *R*^*2*^- the adjusted r-squared

Reference categories: ^A^Yes; ^B^No pregnancy loss; ^C^Miscarriage; ^D^Married; ^E^College/university

## Discussion

The contact of medical personnel with a woman after pregnancy loss is a special type of social interaction involving the exchange of emotions and instruments of action. Both medical staff, because of their competences and the nature of their work, as well as the patient’s relatives belong to the group of people considered significant in the process of adapting to this difficult situation. Assessment of patient expectations and their constant monitoring is conducive to the development of modern medicine and nursing, as well as to meeting the ever-greater requirements towards medical personnel [1, 12].

The type of social support that received the highest score from women after pregnancy loss, both as a result of miscarriage and ectopic pregnancy (EP), was perceived available instrumental, emotional, and actually received social support. The statistical data presented in the present paper is comparable to the results of a study conducted by Konczelska et al. among parents who had experienced the death of a child [16].

Patients after miscarriage and ectopic pregnancy sought support to a lesser extent than respondents who had experienced the death of an older child. This is may be due to the fact that among the respondents studied by Konczelska et al. satisfaction with the support received was assigned the lowest score, while the score for support from individual sources given by the respondents participating in the present study was high [16].

Along with the decreasing scope of social support, the incidence of poor overall health, mental stress, symptoms of anxiety and depression, limited activity, and disability increases [1, 17, 18]. Providing social support and assessing it in the medical community is therefore extremely important [19].

The present study showed a positive correlation between support from both primary (relatives, other patients) and secondary support systems (medical staff, clergy) and satisfaction with quality of life among women in such unique circumstances as pregnancy loss. Moreover, QoL was positively correlated with social support. The present analysis corresponds with the studies by Dębska et al. and Sitjar-Suñer et al., who demonstrated that social support is a key factor for perceived quality of life [20, 21].

In a regression analysis by Bastardo et al., social support was significantly correlated with all HRQL domains except for physical functioning [22]. Our study demonstrated that there was a positive relationship between social support and mental condition, physical condition, and satisfaction with health and quality of life among women after pregnancy loss.

Natural sources of support are considered the most durable and reliable [23]. Interpretation of the results obtained in the course of this study showed that women who had experienced prenatal loss gave the highest scores to natural sources of support, such as their partner and family. Other sources of support that received high scores were the midwife, friends, and the doctor. The present analysis corresponds to the qualitative research conducted by Bellhouse et al. among Australian women after miscarriage, where the partner was identified as the main source of social support [24]. A study conducted by Song et al. indicated that marital closeness mitigated the negative effects of mourning [25]. Social support plays an important role in moving through its successive stages. A low level of support from the family and friends contributes to the emergence of complex mourning reactions. In turn, a weak marital bond or lack of support from the partner are strong factors that intensify the feeling of grief after perinatal loss [6, 7].

By analyzing the data associated with the impact of support on the condition of patients after pregnancy loss, we showed that support from the partner of women with EP and the family of respondents after miscarriage were significantly correlated with their mental state. It should be noted, however, that women who had had a miscarriage assigned slightly higher scores to the level of support received from their partners compared to patients with EP who, in turn, received slightly greater support from their families. It can therefore be assumed that the very initiation of supportive actions on the part of relatives was a factor affecting the condition of patients after pregnancy loss. Moreover, it should be noted that the necessary condition for supportive social interactions to meet the expectations of a person in need is their purposefulness, consistency between the amount of aid provided and the needs and expectations of the recipient, and also mutual (donor-recipient) understanding and mobilization. The effectiveness of these actions also depends on the recipient’s resources, such as self-esteem, competences, conviction about their own effectiveness, social position, sense of control, and coherence [24].

The contact of medical personnel with a woman after pregnancy loss is a special type of social interaction involving the exchange of emotions and instruments of action. Both medical staff, because of their competences and the nature of their work, as well as the patient’s relatives belong to the group of people considered significant in the process of adapting to this difficult situation. Assessment of patient expectations and their constant monitoring is conducive to the development of modern medicine and nursing, as well as to meeting the ever-greater requirements towards medical personnel [1, 12].

The type of social support that received the highest score from women after pregnancy loss, both as a result of miscarriage and ectopic pregnancy (EP), was perceived available instrumental, emotional, and actually received social support. The statistical data presented in the present paper is comparable to the results of a study conducted by Konczelska et al. among parents who had experienced the death of a child [16].

Patients after miscarriage and ectopic pregnancy sought support to a lesser extent than respondents who had experienced the death of an older child. This is may be due to the fact that among the respondents studied by Konczelska et al. satisfaction with the support received was assigned the lowest score, while the score for support from individual sources given by the respondents participating in the present study was high [16].

Along with the decreasing scope of social support, the incidence of poor overall health, mental stress, symptoms of anxiety and depression, limited activity, and disability increases [1, 17, 18]. Providing social support and assessing it in the medical community is therefore extremely important [19].

The present study showed a positive correlation between support from both primary (relatives, other patients) and secondary support systems (medical staff, clergy) and satisfaction with quality of life among women in such unique circumstances as pregnancy loss. Moreover, QoL was positively correlated with social support. The present analysis corresponds with the studies by Dębska et al. and Sitjar-Suñer et al., who demonstrated that social support is a key factor for perceived quality of life [20, 21].

In a regression analysis by Bastardo et al., social support was significantly correlated with all HRQL domains except for physical functioning [22]. Our study demonstrated that there was a positive relationship between social support and mental condition, physical condition, and satisfaction with health and quality of life among women after pregnancy loss.

Natural sources of support are considered the most durable and reliable [23]. Interpretation of the results obtained in the course of this study showed that women who had experienced prenatal loss gave the highest scores to natural sources of support, such as their partner and family. Other sources of support that received high scores were the midwife, friends, and the doctor. The present analysis corresponds to the qualitative research conducted by Bellhouse et al. among Australian women after miscarriage, where the partner was identified as the main source of social support [24]. A study conducted by Song et al. indicated that marital closeness mitigated the negative effects of mourning [25]. Social support plays an important role in moving through its successive stages. A low level of support from the family and friends contributes to the emergence of complex mourning reactions. In turn, a weak marital bond or lack of support from the partner are strong factors that intensify the feeling of grief after perinatal loss [6, 7].

By analyzing the data associated with the impact of support on the condition of patients after pregnancy loss, we showed that support from the partner of women with EP and the family of respondents after miscarriage were significantly correlated with their mental state. It should be noted, however, that women who had had a miscarriage assigned slightly higher scores to the level of support received from their partners compared to patients with EP who, in turn, received slightly greater support from their families. It can therefore be assumed that the very initiation of supportive actions on the part of relatives was a factor affecting the condition of patients after pregnancy loss. Moreover, it should be noted that the necessary condition for supportive social interactions to meet the expectations of a person in need is their purposefulness, consistency between the amount of aid provided and the needs and expectations of the recipient, and also mutual (donor-recipient) understanding and mobilization. The effectiveness of these actions also depends on the recipient’s resources, such as self-esteem, competences, conviction about their own effectiveness, social position, sense of control, and coherence [24].

In the research conducted by Mess et al. among women who had experienced the loss of a child at various stages of pregnancy, most respondents, when asked about the support received in hospital conditions, indicated that this had come from their family and the midwife [26]. In the present analysis, the support provided by the latter also received high scores. The midwife creates, or should create, a special relationship with a patient after pregnancy loss, provides contact with another woman, a person who is a source of safety and support during pregnancy, and who then becomes a witness of the child’s death and the mother’s despair.

Patients after miscarriage and ectopic pregnancy indicated friends as another source of support, and then their gynecologist-obstetrician. 40% of women after pregnancy loss surveyed by Mess et al. indicated their doctor as a supportive person at the hospital [26]. Another important source of social support for patients participating in the diagnostic survey for this study turned out to be other patients in a similar situation who were staying at the hospital at the same time. Bellhouse et al. obtained comparable results among individuals after pregnancy loss [24].

Psychological care received a high score from the women after the loss of a child surveyed by Mess et al.: it ranked third in terms of support provided to the women during hospitalization [26]. The results of the present study did not correspond with Mess et al.’s data. The psychologist was one of the least effective sources of support for patients. This was probably due to the fact that a significant number of women with diagnosed pregnancy loss did not have contact with a psychologist or did not express an opinion on the care provided by this specialist. It should be emphasized that for patients with ectopic pregnancy, psychological care had a significant impact on improving their mental state. Therefore, it should be noted that it is necessary to include psychological care in management algorithms and monitor this process through systematic research in the field of social support among patients after prenatal loss.

Spiritual support, especially in the event of death, where suffering dominates, is very valuable. Religion reduces the feeling of irreversibility of death, explains its meaning, and offers rituals to help process the loss. In the present study support from the clergy received a low score (however, it should be noted that, as in the case of psychological care, a large group of patients did not have contact with a priest or did not express an opinion on this source of support). This result was identical with the data from the analysis conducted by Mess et al., where support from a priest was received by a small group of patients after perinatal loss (approx. 3%) [26]. A literature review also shows that religious communities constitute beneficial sources of support, and religion is associated with increased perception of social support [7]. The present analysis showed a positive correlation between clerical support and satisfaction with the quality of life in women who had experienced obstetric failure.

In the regression model, the percentage of explained variance in QoL scores was relatively low, which suggests that the variable is explained by other predictors than those we considered. Other researchers have indicated that women after pregnancy loss, compared to those who have never had such an experience, report poorer QoL in terms of psychological condition, social functioning, or health, among other areas [27–29]. Notably, however, relatively few studies have addressed the factors determining QoL in women after miscarriage or ectopic pregnancy. The woman’s relationship with her partner and loved ones has been identified as one of the most important factors in her QoL. Its impact is not consistent, though, as e.g. the partner may provide support in some cases, but in others, the pregnancy loss may lead to a breakdown of the relationship or marriage [7, 30, 31]. Another important variable that helps better understand the QoL in women after pregnancy loss is the time that has passed since the event. As it turns out, women after pregnancy loss experience a transient decline in overall satisfaction with life and a lasting deterioration of satisfaction in a number of specific areas, e.g. social life [32].

One of the current challenges facing the healthcare system is the appropriate response to patient expectations. The quality of the services provided should be assessed constantly in order to make improvements by designating and implementing appropriate strategies. Patient expectations, as well as their experiences and opinions on maternity care, should constitute an important message for both health care employees and administrators, while ensuring the psychosocial comfort of patients, in addition to effective treatment and care, should be one of the priorities in medical personnel’s daily work. Raising public awareness of the impact of pregnancy loss on psychophysical health seems necessary to shed more light on this profoundly personal experience, as well as to better understand and provide proper support to people affected by prenatal loss.

## Limitations of the review

### Strengths and limitations of this study

The advantage of our work is the size of the study group (610 people) and the fact that our questionnaire was delivered to each respondent in person. It should also be emphasized that the study utilized a standardized tool, which allows other authors studying the issue of miscarriage to compare research results and explore the subject, as well as to monitor changes. The available studies conducted among women after miscarriage are usually performed several weeks or months after the event. The present study was carried out during hospitalization, as the struggle with this difficult situation in most cases begins in the hospital.

Another important aspect of this study is the attention paid to patients diagnosed with ectopic pregnancy, which is most often treated as a life-threatening condition, while the fact of losing a child is considered secondary. Although there are studies on the negative impact of ectopic pregnancy on women’s mental health, these patients are often not taken into account in the procedures implemented after pregnancy loss. Conducting research on social support in this group of women and recognizing them as individuals experiencing the loss of a child, not just clinical cases, is therefore extremely important and can significantly affect their overall biopsychosocial well-being.

One of the difficulties related to this study was delivering questionnaires in person during a hospital stay. This resulted from the duration of the stay (3–6 days), during which intensive diagnostic and therapeutic procedures were performed. Therefore, in order to ensure the highest quality and credibility of the research, information on the stage of treatment, hospitalization time, and opinions on the clinical condition of the women was obtained from medical personnel before contact with each respondent. The study is also limited by its cross-sectional nature, which does not allow for the establishment of cause-effect relationships between social support and subjective assessment of psychophysical condition, health, and satisfaction with quality of life. Another limitation is the fact that in our study psychophysical condition and health were assessed using an original survey questionnaire. Moreover, the study did not include an evaluation of individual characteristics and reactions, which could have affected the patients’ perception of their psychophysical condition and of the support received.

In the present study, we considered the same set of variables explaining women’s physical condition, psychological condition, quality of life and satisfaction with health after pregnancy loss. For quality of life, only a small percentage of variance was accounted for by these variables. In the light of the available literature, it seems that factors such as the quality of a patient’s relationship with her partner, her relationship with her family, or time since the event may be crucial to the understanding of QoL in women after pregnancy loss.

## Conclusions

Women hospitalized due to miscarriage and ectopic pregnancy assigned high scores to the level of perceived available instrumental, emotional, and actually received social support. The assessment of their psychophysical condition, and satisfaction with health and quality of life was determined by sociodemographic factors and obstetric history. Social support received from relatives, hospital staff, and patients in a similar situation correlated with the assessment of the condition of women after pregnancy loss.

The present study indicates that health and social policy should be implemented in all countries with greater awareness by introducing training for medical personnel in the field of professional support in difficult situations related to motherhood, management algorithms and control of their implementation, as well as by providing appropriate psychological help, which is not received by patients to a sufficient degree. Proper conduct of medical personnel and support from relatives may contribute to the optimization of obstetric care and minimization of negative effects on the mental health of patients after pregnancy loss and positively affect their psychophysical condition, health, and quality of life.

## Supplementary Information

**Additional file 1 **: **Supplementary file 1**. Original author’s survey questionnaire.

**Additional file 2 **: **Supplementary file 2**. Survey questionnaire in English.

## Data Availability

All data generated or analyzed during this study are included in this published article.

## Abbreviations

BSSS: Berlin Social Support Scales
BSSS I Emo: Perceived available Emotional Support
BSSS I Instr: Perceived available Instrumental Support
BSSS II: Need for Support; BSSS III - Support Seeking
BSSS IV: Actually Received Support
BSSS V: Protective Buffering Support
EP: Ectopic pregnancy
M: Mean
Me: Median
QoL: Quality of Life
SD: Standard deviation
